# Disparities in Pediatric Concussion Outcomes and Family Burden by Neighborhood Opportunity

**DOI:** 10.3390/bs16081287

**Published:** 2026-07-28

**Authors:** Karen Nakhla, Dana Waltzman, Lindsay D. Nelson, Anthony P. Kontos, Danny G. Thomas

**Affiliations:** 1 School of Medicine, Wayne State University, 540 E Canfield St., Detroit, MI 48201, USA; karen.nakhla@wayne.edu; 2 Department of Physical Medicine & Rehabilitation, Indiana University School of Medicine, Indianapolis, IN 46202, USA; dwaltzma@iu.edu; 3 Departments of Neurosurgery and Neurology, Medical College of Wisconsin, 9200 W Wisconsin Ave., Milwaukee, WI 53226, USA; linelson@mcw.edu; 4 Department of Orthopaedic Surgery, University of Pittsburgh School of Medicine, 3471 Fifth Ave., Pittsburgh, PA 15213, USA; 5 Department of Pediatrics, Medical College of Wisconsin, Children’s Corporate Center, 999 N. 92nd St., Milwaukee, WI 53226, USA

**Keywords:** concussion, child opportunity index, disparities

## Abstract

Mild traumatic brain injury (mTBI) is a common cause for pediatric emergency department visits and can impose substantial burdens on families, including missed work and childcare needs. Socioeconomic context may influence both healthcare use and these downstream impacts. This study examined the association between Child Opportunity Index (COI), a neighborhood-level measure of socioeconomic status, and healthcare utilization and indirect costs following pediatric concussion. In a secondary analysis of a multisite randomized controlled trial of patients with acute (<72 h) concussion, participants were categorized into high, middle, and low COI groups. Outcomes included follow-up care, caregiver missed workdays, childcare needs, and indirect costs, analyzed using multivariable regression models. Participants from lower COI neighborhoods were more likely to present to the emergency department, with 93.02% compared to 71.43% in higher COI groups (*p* = 0.005); had higher symptom scores at 14 days, 28.69 versus 16.64 (*p* = 0.03); and were less likely to be seen by concussion specialists, 6.98% versus 28.57% (*p* = 0.005). They also had lower odds of attending follow-up within 14 days (OR = 0.36, *p* = 0.015) and higher caregiver burden, including more missed workdays and childcare needs. Although total indirect costs did not differ significantly for low COI groups after adjustment, middle COI participants had lower costs than high COI ones. Overall, disparities were observed in care patterns and caregiver burden despite similar recovery outcomes. These findings suggest that expanding access to affordable concussion follow-up through school-based programs, telehealth services, transportation assistance, and flexible scheduling may help reduce caregiver burden and improve continuity of care for children from lower COI neighborhoods following pediatric mTBI.

## 1. Introduction

Mild traumatic brain injury (mTBI), commonly known as concussion, is a leading cause of pediatric injury, which is estimated to affect 1.5 million children in the United States annually (Waltzman et al., 2025). Concussions are associated with a variety of symptoms, including headache, dizziness, and blurred vision (Mayo Clinic, 2025). Concussions also create a significant financial strain on families and caregivers. This includes direct costs such as medical expenditures and indirect costs such as missed school days and caregiver workdays as well as lost work productivity (Nelson et al., 2019; Shen et al., 2021).

Increasing attention has been focused on how socioeconomic factors influence both concussion symptom recovery and overall cost of care (Corwin et al., 2026; Graves et al., 2015). Prior research has identified how disparities affect concussion diagnosis and the utilization of healthcare services across racial and socioeconomic groups. National and multicenter studies have found that those from lower socioeconomic backgrounds and historically marginalized racial and ethnic groups are less likely to receive concussion diagnoses, specialty referrals, and outpatient follow-up care, while being more likely to utilize emergency department services for concussion-related care (Corwin et al., 2024; Loftin et al., 2023; Shen et al., 2021). These findings suggest that socioeconomic factors may play a significant role in access to concussion evaluation and longitudinal management.

More broadly, these findings reflect a consistent pattern in which socioeconomic disadvantage is associated with reduced access to specialty follow-up care and less intensive management of concussions, even when injury severity is similar (Cook et al., 2024; MacEachern et al., 2024; Powers et al., 2024; Wing et al., 2024). Such disparities likely arise from differences in insurance coverage, healthcare access, caregiver resources, and the ability to navigate follow-up systems after acute injury. Collectively, these fall within the broader framework of social determinants of health (SDOH), which encompass the structural and environmental conditions that shape health, including healthcare access, neighborhood resources, housing stability, and transportation (*Healthy people 2030: Social determinants of health*, n.d.). To better capture these multidimensional influences, neighborhood-level measures such as the Child Opportunity Index (COI) have been developed. COI integrates indicators across environmental, educational, health, and socioeconomic domains to reflect the structural conditions that shape child health outcomes (Noelke et al., 2020), offering a more comprehensive assessment of SDOH than traditional socioeconomic measures alone.

Emerging evidence supports the relevance of COI in pediatric concussion. For example, Corwin et al. (2026) demonstrated that lower neighborhood opportunity was associated with differences in healthcare utilization, including reduced access to specialty concussion care and follow-up services, even when accounting for injury-related factors. These findings suggest that neighborhood context may influence not only access to care but also how recovery is managed over time.

However, prior work has largely focused on isolated aspects of care, such as utilization patterns (Corwin et al., 2026; Graves et al., 2019), while less is known about how neighborhood opportunity relates simultaneously to symptom burden, recovery trajectories, and the broader impact on families, including indirect costs and caregiving demands. This gap limits a more comprehensive understanding of how structural factors shape the full recovery experience following pediatric concussion.

Building on this framework, the aim of this exploratory study is to examine the association between neighborhood child opportunity (COI) and pediatric concussion, including clinical outcomes (symptom burden and recovery), healthcare utilization (including specialist access and follow-up attendance), and family burden. We hypothesized that children from lower-opportunity neighborhoods would experience greater symptom burden, poorer recovery, reduced healthcare utilization, and greater family burden than children from higher-opportunity neighborhoods. Understanding these relationships may help identify structural barriers to optimal recovery and inform interventions aimed at improving access and reducing burden in lower-opportunity communities.

## 2. Materials and Methods

This study was a secondary analysis of a multicenter, prospective randomized controlled trial (Active Injury Management Trial; ClinicalTrials.gov identifier NCT03869970) (Thomas et al., 2025). Participants were aged 11–24 years and diagnosed with acute mild traumatic brain injury (mTBI) within 72 h of injury at a pediatric emergency department or outpatient sports concussion clinic. Exclusion criteria included a prior concussion within 6 months, moderate or severe TBI, a history of major psychiatric or neurological disorders, developmental delay, vestibular disorders, concurrent orthopedic injury, substance abuse, hospital admission, lack of smartphone access, the inability to provide consent, or the inability to complete study procedures. This study was approved by the Medical College of Wisconsin Institutional Review Board (IRB#PRO33221).

### 2.1. Outcome Measures

Neighborhood Opportunity: The Child Opportunity Index (COI) is a composite measure derived from 29 indicators across education, health and environment, and social and economic domains. Participants were categorized into three COI groups based on residential neighborhood: low (1–40), middle (41–59), and high (60–100). These categories were created by collapsing the standard five COI quintiles into three groups (combining the two lowest and two highest quintiles). Acevedo-Garcia et al. (2020) Given the relatively small sample size, particularly within individual quintiles, this approach was chosen to improve statistical power and clinical interpretability while preserving the established COI thresholds.

Symptom Severity: The Post-Concussion Symptom Scale (PCSS) (Karr et al., 2023) measures 22 self-reported symptoms from 0 (none) to 6 (severe). A higher score indicates more symptom burden. The PCSS was assessed at 5 days, 14 days, 1 month, and 2 months.

Functional Recovery: Functional recovery was assessed by full symptom resolution and percent return to school and sports. Symptom resolution was determined by an affirmative answer to the question, “Have your child’s symptoms resolved?” and was assessed at 1 and 2 months. Return to school was determined by an affirmative answer to the question, “Did your child return to school?” and was assessed at 14 days. Return to sports was determined by an affirmative answer to the question, “Has your child returned to organized sports/recreation?” and was assessed at 1 and 2 months.

Healthcare Utilization: Parents were asked questions about the number and type of follow-up visits (at 0 to 14 days, 14 days to 1 month, and 1 to 2 months) to specialty physicians and the number of days to the first follow-up visit (at 0 to 14 days).

Family Burden: Parents were asked questions about indirect costs (at 0 to 14 days), missed workdays (paid and unpaid days at 0 to 14 days), the number of childcare days needed (at 0 to 14 days), and the number of school days missed (at 1 and 2 months). Indirect costs were calculated as the sum of the responses to 2 questions: (1) “Approximately how much have you spent out of your own pocket as a result of your child’s concussion/injury on insurance copayments/deductibles?” And (2) “Approximately how much have you spent out of your own pocket as a result of your child’s concussion/injury on medication or health services?”

Parental Perception: Parents were asked questions about their perceptions of their child’s recovery, symptoms, and problems post injury. Responses were measured on a Likert scale with options that included: strongly agree, agree, unsure, disagree, and strongly disagree. Responses to “strongly agree” and “agree” are reported.

### 2.2. Statistical Analysis

Descriptive and bivariate statistics were calculated to describe the overall sample and the sample stratified by COI over time by demographics, symptom presentation, recovery, and family burden. These analyses were conducted separately at each study time point and are presented as unadjusted descriptive and bivariate comparisons (Table 1, Table 2, Table 3, Table 4 and Table 5). Separate multivariable regression models were used to assess associations between COI group and each outcome of interest. The multivariable models simultaneously incorporated all available time points, where applicable, and adjusted for relevant demographic and clinical covariates (Table 6). All models were adjusted for by relevant demographics (age, sex, insurance, site, and prior concussion). Specifically, a baseline constrained linear mixed model was run to determine if PCSS trajectory over time differed by COI group and included fixed effects for age, sex, insurance, site, prior concussion, time point, and COI time point interaction, and a random participant effect with equal baselines was assumed (Coffman et al., 2016). Time was modeled as a categorical variable with five assessment time points (baseline, 5 days, 14 days, 1 month, and 2 months). The baseline was specified as the reference level. Unstructured and AR(1) covariance structures were compared for model selection using the Akaike Information Criterion and the Bayesian Information Criterion with the unstructured covariance matrix selected for a better model fit. Logistic regressions were conducted for the outcomes of symptom resolution by 1 month and follow-up visits by 14 days. A combined two-part marginal analysis (logistic and gamma regression) using 1000 bootstrap replications was run for the outcome of total indirect costs by 14 days. Poisson regression was used for the outcome of number of follow-up visits by 14 days, while a negative binomial regression was used for number of work days missed and childcare days needed by 14 days. Addition analyses included a sensitivity analysis modeling COI as a continuous variable and characterizing missing data (Supplemental Table S1). Analyses were run in SAS 9.4 (SAS Institute, Cary, NC, USA).

## 3. Results

Characteristics of the overall sample and by COI group are presented in Table 1. Significant differences in demographic, socioeconomic, and clinical characteristics were observed across COI groups. Racial distribution varied, with participants from low COI neighborhoods more likely to be Black (56.06%) compared to middle (15.38%) and high COI groups (6.98%), and less likely to be White (37.88% vs. 84.62% and 90.70%, respectively). Similarly, Hispanic ethnicity was more prevalent in the low COI group (17.19%) compared to middle (5.26%) and high COI groups (7.14%). Participants in low COI neighborhoods were also less likely to have commercial insurance (57.58%) compared to middle (87.18%) and high COI groups (95.35%). Conversely, Medicaid HMO coverage was more common in the low COI group (36.36%) compared to middle (10.26%) and high COI groups (2.33%), and Medicaid coverage was present only in the low COI group (3.03%) and absent in the high COI group. Caregiver education differed significantly between low and high COI groups. Caregivers in low COI neighborhoods were less likely to have a bachelor’s degree or higher (21.54% vs. 55.91%) compared to those in high COI neighborhoods. Differences in healthcare utilization at presentation were also observed. Participants from low COI neighborhoods were more likely to present to the emergency department (93.02%) compared to high COI groups (71.43%), while those from high COI neighborhoods were more likely to be seen in concussion clinics (28.57% vs. 6.98% and 10.34% for low and middle COI, respectively). Finally, time from injury to study enrollment differed significantly, with participants from low COI neighborhoods enrolling earlier (0.52 days) compared to those from middle COI neighborhoods (0.87 days).

**Figure 1:**
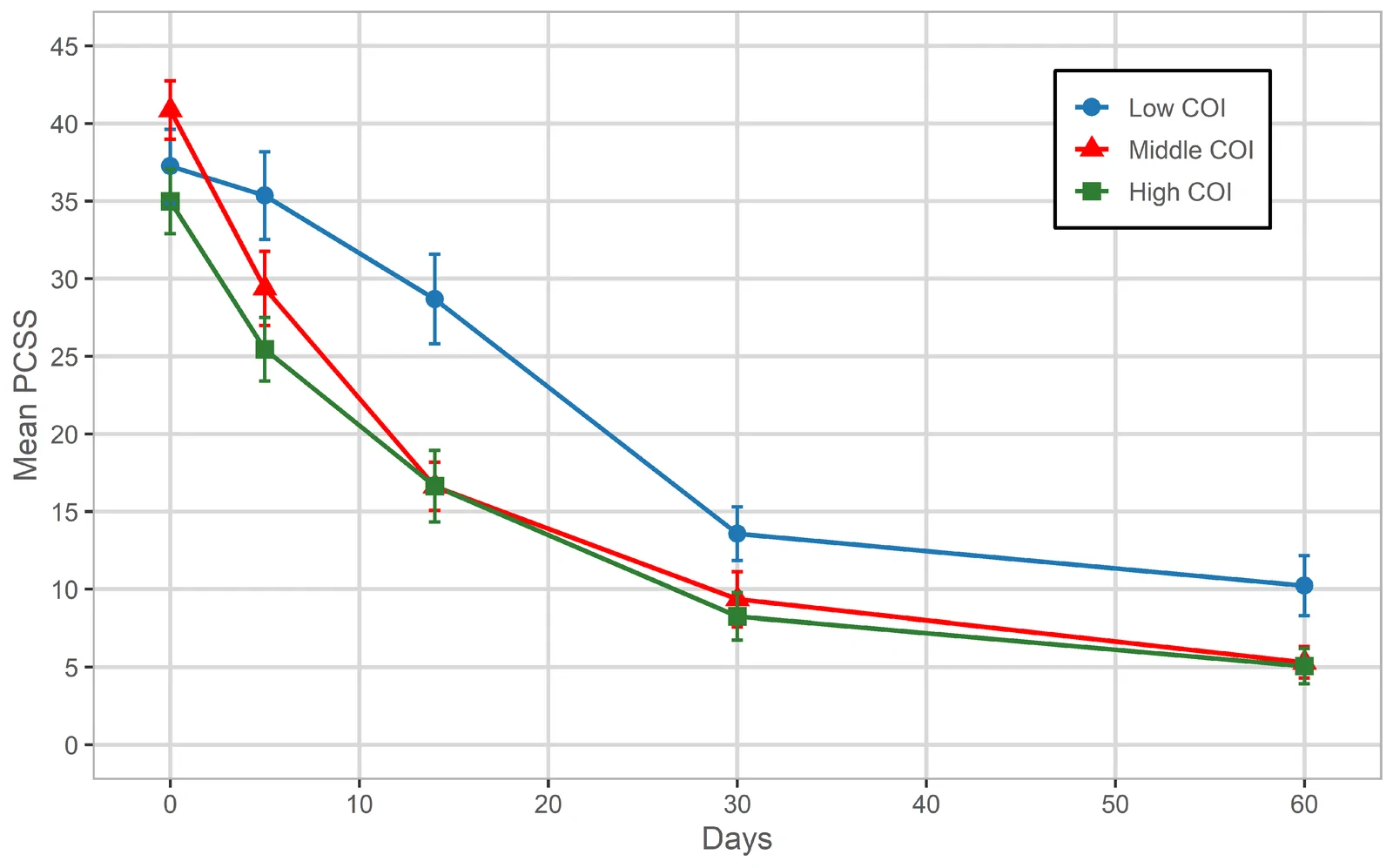
The mean difference in PCSS over time between COI groups. The sample size at each time point is as follows: baseline (day 0) = 232, 5 days = 208, 14 days = 155, 1 month (30 days) = 191, and 2 months (60 days) = 181. The errors bars represent standard errors.

PCSS at the baseline, 1 month, and 2 months did not differ significantly across COI groups (Table 2 and Figure 1). However, at 14 days post-injury, participants from low COI neighborhoods had a significantly higher symptom burden compared to those from high COI neighborhoods (28.69 vs. 16.64). Additionally, by 1 month post-injury, a significantly greater proportion of participants from low (36.17%) and middle COI neighborhoods (39.39%) had fully returned to sports compared to those from high COI neighborhoods (10.94%) (*p* = 0.03). There were no significant differences between COI groups for symptom resolution and return to sports.

**Figure 2:**
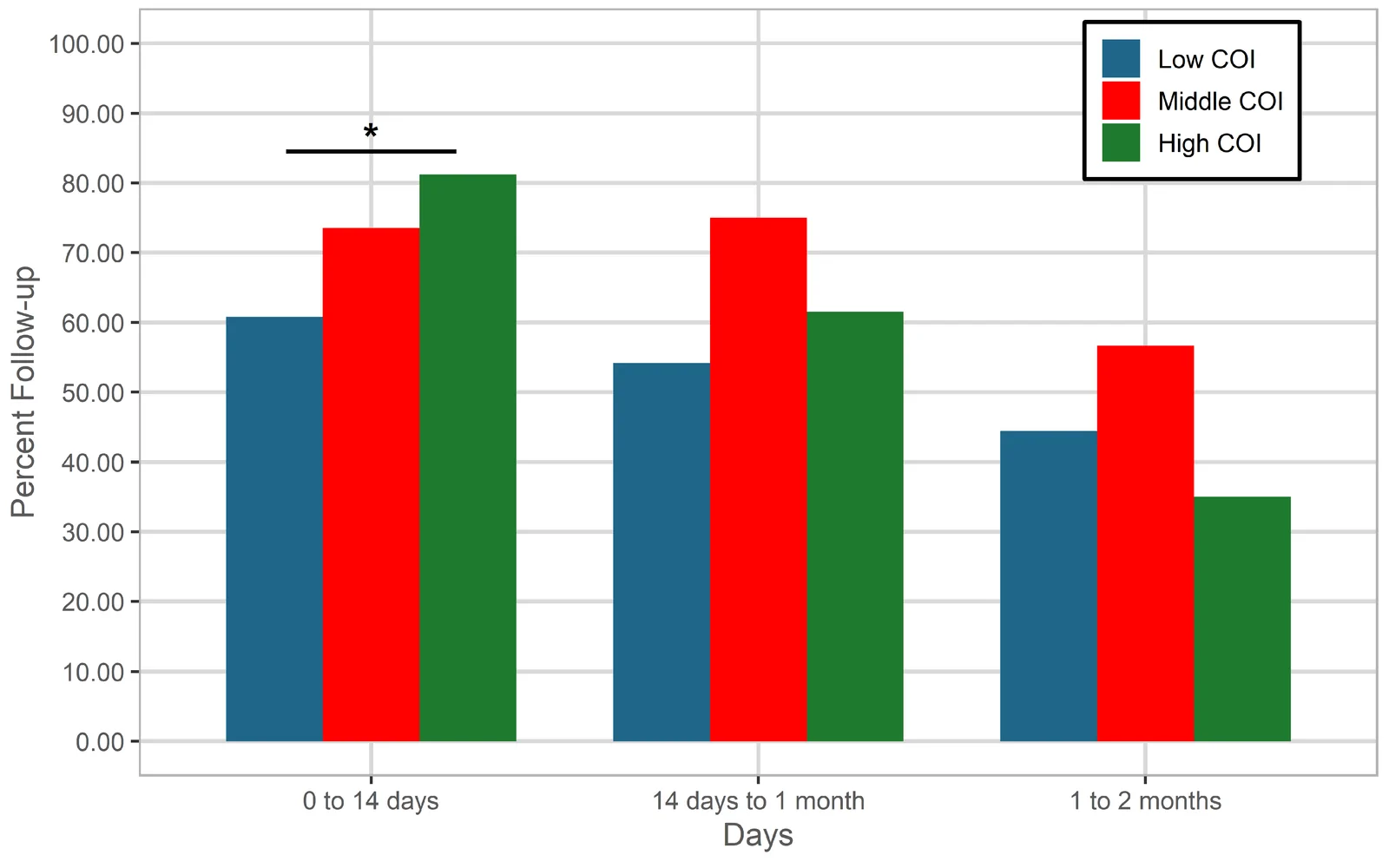
Follow-up in healthcare utilization over time between COI groups. The asterixis denotes significant differences, *p* < 0.05.

Healthcare utilization within the first 14 days post-injury differed significantly by COI (Table 3 and Figure 2). Participants from low COI neighborhoods were less likely to have any follow-up visit compared to those from high COI neighborhoods (60.78% vs. 81.20%, *p* = 0.02). Similarly, children from low COI groups were less likely to be evaluated by a sports medicine specialist (20.00% vs. 46.96%, *p* = 0.003) and less likely to receive physical therapy services (2.04% vs. 12.50%, *p* = 0.04) within 14 days. All other indicators of healthcare utilization were not significant between COI groups.

Caregiver burden within the first 14 days post-injury differed significantly by COI (Table 4). Participants from low COI neighborhoods had a higher mean number of missed workdays compared to those from high COI neighborhoods (3.38 vs. 1.70 days, *p* = 0.02). Similarly, families in the low COI group required more childcare support (1.56 vs. 0.32 days, *p* = 0.01). There was no significant difference in total indirect costs or in school days missed at 1 and 2 months post-injury between groups.

Parental perception of injury severity did not differ significantly across COI groups (Table 5). However, care-seeking behavior varied substantially (Table 6). After adjustment, participants from low COI neighborhoods were significantly less likely to engage in follow-up care compared to those from high COI neighborhoods, demonstrating lower rates of any follow-up visits (OR = 0.36, 95% CI = 0.16–0.82, *p* = 0.015) within the first 14 days post-injury. Additionally, in two-part marginal analyses, the adjusted predicted mean 14-day indirect cost was similar between low and high COI groups ($268.63 vs. $248.72), with no significant difference (difference $19.91, 95% CI −$12.20 to $54.17, *p* = 0.21). However, participants in middle COI neighborhoods had significantly lower indirect costs compared to those in high COI neighborhoods (difference −$69.47, 95% CI −$104.75 to −$35.17, *p* < 0.001). Healthcare utilization patterns differed despite similar total visit counts. COI was not associated with total visits at 14 days in adjusted Poisson models (IRR = 0.75, 95% CI = 0.52–1.11, *p* = 0.13). However, participants from low COI neighborhoods had significantly lower odds of attending a follow-up visit at 14 days compared to those from high COI neighborhoods (OR = 0.36, 95% CI = 0.16–0.82, *p* = 0.015). Caregiver burden was significantly higher in lower COI groups. In adjusted models, participants from low COI neighborhoods missed more workdays (IRR = 2.01, 95% CI = 1.28–3.17, *p* = 0.003), with an adjusted mean of 3.7 days compared to 1.8 days in high COI groups. They also required more childcare support (IRR = 4.17, 95% CI = 1.65–10.51, *p* = 0.003), with an average of 1.93 days versus 0.46 days among high COI participants. COI was not associated with changes in symptom burden over time and symptom resolution at 1 month. COI was also not associated with other indicators of cost (i.e., overall indirect cost burden or cost magnitude). In a sensitivity analysis, COI was modeled as a continuous predictor (Supplemental Table S2). The direction and statistical significance of the associations were unchanged, suggesting that the findings were robust to the specification of COI.

## 4. Discussion

This exploratory study analyzed the association between COI and concussion recovery (symptom burden, healthcare utilization, and indirect costs) following pediatric concussion. Our findings demonstrate that a lower COI was associated with reduced odds of timely follow-up, decreased access to concussion specialists, higher symptom burden at 14 days, and significantly greater indirect costs and burden to families. Notably, these symptom differences were not observed at later time points, suggesting that disparities may be most pronounced during the early recovery phase.

The community- and socioeconomic-level disparities we observed in healthcare utilization align with previous work. Prior research has found that children from lower COI neighborhoods are more likely to present to the emergency department and have reduced access to primary and specialty care compared with those from higher-opportunity areas (Corwin et al., 2024). This is in line with our study findings, as our results demonstrate that participants from low COI neighborhoods were more likely to present to the emergency department, while those from high COI neighborhoods were more likely to be seen in concussion clinics. More recent population-level data further demonstrate that patients from socioeconomically marginalized communities that initially sought care in the ED as opposed to outpatient clinics were also significantly less likely to complete follow-up care after concussion (Tillmann & Haas, 2026). This is consistent with our findings, which highlight that children from lower COI neighborhoods were less likely to attend a follow-up visit at 14 days, despite similar overall visit rates. This suggests that differences are not due to total healthcare use but rather reduced engagement with structured, outpatient follow-up care, which is critical for concussion management. Similarly, prior work (Kersten et al., 2018; Krager et al., 2021) has demonstrated that lower-opportunity environments are associated with barriers to accessing longitudinal and specialty care. COI domains, such as economic resources, employment flexibility, transportation access, and health literacy, likely contribute to these patterns (Noelke et al., 2020). For example, limited job flexibility or lack of paid leave may hinder caregivers’ ability to attend scheduled visits, while differences in educational resources may influence understanding of the importance of follow-up.

However, studies examining the broader impact of socioeconomic conditions on caregiver burden remain limited, despite evidence that pediatric traumatic brain injury is associated with increased family disruption, missed work, and financial strain (Ganesalingam et al., 2008). Our findings provide evidence that caregiver burden differed substantially by COI, even with the similarity in long-term clinical outcomes. Families in lower COI neighborhoods had more than double the amount of missed workdays compared to families in high COI groups. They also required more than four times more childcare support during the recovery period. These findings point to a disproportionate burden placed on families from low resource neighborhoods, reflecting reduced flexibility and fewer available support systems. Although total indirect costs did not differ significantly between low and high COI groups in adjusted analyses, this could be due to variability and limited power rather than true equivalence. Notably, the nature of family burden differed: families from lower COI neighborhoods experienced greater disruption in terms of time and caregiving demands, which may not be fully captured by monetary estimates. The significantly lower indirect costs observed in middle COI groups compared to high COI groups further suggest a more complex, non-linear relationship, potentially reflecting differences in employment flexibility or access to social support networks.

Contrary to our initial hypothesis, low COI was not associated with longer-term clinical outcomes. Post-concussion symptom scores did not differ significantly across COI groups at the baseline, 1 month, or 2 months post-injury. However, at 14 days post-injury, participants from low COI neighborhoods demonstrated a significantly higher symptom burden compared with those from high COI neighborhoods. Despite this early difference, symptom trajectories converged over time, with no significant between-group differences observed at later time points.

These findings suggest many important points. First, children from lower COI neighborhoods experienced greater symptom burden during the early recovery period but were less likely to receive follow-up care. This indicates a mismatch between clinical need and healthcare utilization, where those presenting with greater early symptoms had reduced access to ongoing evaluation and management. Reduced follow-up care during a period of higher symptom burden may contribute to a more fragmented and potentially more burdensome recovery experience, even when eventual clinical recovery is comparable. Second, the apparent disconnect between similar clinical outcomes and unequal recovery experiences is a key finding. As COI encompasses multiple domains, including education, environment, and social and economic conditions, higher COI environments may provide greater access to resources that facilitate recovery, such as flexible employment, stable housing, access to childcare, and greater healthcare navigation support. In contrast, lower COI environments may impose competing priorities and structural constraints that limit follow-up engagement and amplify the day-to-day burden of managing a child’s recovery.

These findings have important implications for clinical practice and health policy. First, they suggest that standard clinical outcome measures may underestimate the true impact of disparities, as they fail to capture differences in access, continuity of care, and caregiver burden. Second, disparities in concussion are less focused on the differences in recovery and more about the conditions that shape the recovery process. Therefore, although outcomes may look similar, the path to recovery and the cost of getting there remains unequal. Third, they underscore the need for targeted interventions during the early recovery period, when disparities appear most pronounced. Potential strategies to help provide greater equity include improving access to follow-up through telehealth, enhancing care coordination, providing clear discharge education, and implementing support services that reduce logistical barriers for families in lower-opportunity settings (Yeates et al., 2023).

This study should be interpreted in the context of several limitations. First, participants were recruited from pediatric emergency departments and outpatient sports concussion clinics within 72 h of injury, which may limit generalizability to children who do not seek early medical care or who are managed exclusively in primary care or community settings. In addition, several eligibility criteria may further restrict generalizability. Exclusion of individuals with a prior recent concussion, moderate or severe TBI, major psychiatric or neurological conditions, developmental delay, vestibular disorders, concurrent orthopedic injury, substance use, or lack of smartphone access likely resulted in a more clinically and socially stable cohort than the broader pediatric concussion population. As a secondary analysis of a randomized controlled trial, this study was limited to variables collected for the primary trial objectives. Consequently, important family-level factors, including family structure and household composition, and individual-level factors, such as sleep and other determinants of recovery, were either unavailable or not collected in sufficient detail for inclusion in the present analyses. Moreover, outcome measures relied heavily on parent- and self-reported assessments, including symptom severity, healthcare utilization, functional recovery, and family burden. These measures may be subject to recall bias and reporting variability, particularly for indirect costs, missed work, and childcare needs. Finally, as with most longitudinal concussion cohorts, loss to follow-up and missing data may introduce biases despite analytic adjustments.

Future research should aim to disaggregate COI into its individual domains (education, health/environment, and social/economic factors) to determine which components most strongly drive differences in follow-up care and caregiver burden. This would allow for more targeted, mechanism-based interventions. Future research should also investigate potential strategies to reduce disparities, including telehealth follow-up, care navigation programs, school-based concussion management, and policies that improve access to paid leave or childcare support. These approaches were not directly evaluated in the present study and warrant further investigation to determine their feasibility, scalability, cost-effectiveness, and implementation across diverse healthcare settings. Randomized or implementation-focused studies would help determine whether improving access to follow-up care can meaningfully reduce caregiver burden, even if symptom trajectories remain unchanged.

## 5. Conclusions

While pediatric concussion recovery outcomes appear similar across COI groups, substantial disparities exist in healthcare utilization and caregiver burden, particularly in the early post-injury period. These findings suggest that inequities in pediatric concussion are less about differences in recovery and more about differences in the conditions under which recovery occurs, highlighting the need for interventions that address structural barriers and help support families beyond the clinical setting.

## Figures and Tables

**Table 1:** Cohort characteristics at enrollment by COI stratum: unadjusted descriptive and bivariate results.

Characteristic	Overall (N = 234)		Low COI (n = 66)		Middle COI (n = 39)		High COI (n = 129)		Statistic	*p*-Value	Statistically Significant Between COI Levels ^#^
	N	Mean/%	N	Mean/%	N	Mean/%	N	Mean/%			
Age (years)									0.18	0.83	
Mean (SD)	234	14.46 (2.23)	66	14.38 (1.98)	39	14.65 (2.69)	129	14.45 (2.22)			
Sex; n (%)									1.37	0.50	
Male	121	51.71	34	51.52	17	43.59	70	54.26			
Female	113	48.29	32	48.48	22	56.41	59	45.74			
Race; n (%)									69.80	<0.0001 ^†^	
White	175	74.79	25	37.88	33	84.62	117	90.70			^a,b^
Black	52	22.22	37	56.06	6	15.38	9	6.98			^a,b^
Other ^	6	2.56	3	4.55	0	0.00	3	2.33			
Not reported	1	0.43	1	1.52	0	0.00	0	0.00			
Ethnicity; n (%)									7.83	0.0979 ^†^	
Hispanic	22	9.65	11	17.19	2	5.26	9	7.14			^a,b^
Non-Hispanic	200	87.72	50	78.13	35	92.11	115	91.27			
Unknown/Not reported	6	2.63	3	4.69	1	2.63	2	1.59			
Primary language; n (%)									4.97	0.08	
English	233	99.57	65	100.00	38	97.44	128	100.00			
Other	1	0.43	0	0.00	1	2.56	0	0.00			
Insurance type; n (%) public									52.45	<0.0001 ^†^	
Commercial	195	83.33	38	57.58	34	87.18	123	95.35			^a,b^
Medicaid	2	0.85	2	3.03	0	0.00	0	0.00			^b^
Medicaid HMO	31	13.25	24	36.36	4	10.26	3	2.33			^a,b^
Medicare/Other Government	1	0.43	0	0.00	0	0.00	1	0.78			
None (self-pay)	5	2.14	2	3.03	1	2.56	1	0.78			
Caregiver education; n (%)									29.00	<0.0001 ^†^	
High school or less	41	17.83	20	30.77	8	21.05	13	10.24			^b^
Some college	74	32.17	28	43.08	14	36.84	32	25.20			^b^
Bachelor’s degree or higher	100	43.48	14	21.54	15	39.47	71	55.91			^b^
Unknown	15	6.52	3	4.62	1	2.63	11	8.66			
Child Opportunity Index; n (%)									-	-	
Low	66	28.21	-	-	-	-	-	-			
Middle	39	16.67	-	-	-	-	-	-			
High	129	55.13	-	-	-	-	-	-			
Care site; n (%)									10.75	0.0046 ^†^	
ED	136	80.00	40	93.02	26	89.66	70	71.43			^b,c^
Concussion clinic	34	20.00	3	6.98	3	10.34	28	28.57			^b,c^
Mechanism of injury; n (%)									16.49	0.0866 ^†^	
Sports	84	36.21	15	23.08	14	35.90	55	42.97			^b^
Fall	69	29.74	21	32.31	17	43.59	31	24.22			^c^
Motor vehicle collision	12	5.17	6	9.23	1	2.56	5	3.91			
Pedestrian vs. motor vehicle	2	0.86	1	1.54	0	0.00	1	0.78			
Assault	17	7.33	7	10.77	3	7.69	7	5.47			
Other	48	20.69	15	23.08	4	10.26	29	22.66			
Prior concussion; n (%)									4.23	0.12	
Yes	60	25.75	13	20.00	7	17.95	40	31.01			
No	173	74.25	52	80.00	32	82.05	89	68.99			
Baseline PCSS									1.01	0.37	
Mean (SD)	232	36.60	64	37.25 (25.68)	39	40.87 (20.19)	129	34.99 (22.43)			
Time from injury to enrollment (days)									3.42	0.03	^b^
Mean (SD)	234	0.74 (0.91)	66	0.52 (0.85)	39	0.69 (0.80)	129	0.87 (0.96)			

^ Includes American Indian/Alaskan Native, Unknown, Asian, and Native Hawaiian/Pacific Islander. ^†^ Significant linear trend (*p* < 0.05). ^#^ For post hoc tests, pairwise comparisons of column proportions were computed. ^a^ Statistically significant difference (*p* < 0.05) between low COI and middle COI. ^b^ Statistically significant difference (*p* < 0.05) between low COI and high COI. ^c^ Statistically significant difference (*p* < 0.05) between middle COI and high COI.

**Table 2:** Symptom severity and functional recovery over time by COI stratum: unadjusted descriptive and bivariate results.

Outcome	Time Point	Overall		Low COI		Middle COI		High COI		Statistic	*p*-Value	Statistically Significant Between COI Levels
		N	Mean/%	N	Mean/%	N	Mean/%	N	Mean/%			
PCSS score; mean (SD)	5 days	208	28.72 (23.86)	53	35.36 (28.94)	36	29.39 (24.13)	118	25.46 (20.71)	2.61 ^ψ^	0.08	
	14 days	155	19.37 (24.40)	36	28.69 (28.86)	24	16.63 (15.85)	94	16.64 (23.78)	3.46	0.03	^b^
	1 month	191	9.80 (15.76)	49	13.57 (17.20)	33	9.36 (17.01)	108	8.26 (14.58)	1.94	0.15	
	2 months	181	6.43 (13.12)	47	10.23 (18.56)	32	5.31 (9.50)	101	5.07 (10.68)	2.67	0.07	
Symptom resolution; n (%) yes	1 month	135	73.77	30	63.83	25	80.65	80	76.92	^‡^	0.35	
	2 months ^	30	65.22	7	58.33	7	87.50	15	60.00	^‡^	0.64	
Return to school; n (%) full in person	14 days	132	90.41	26	81.25	23	95.83	82	92.13	^‡^	0.17	
Return to sports; n (%) fully returned	1 month	92	49.73	17	36.17	13	39.39	62	59.62	10.94	0.03	^b,c^
	2 months ^	28	56.00	5	41.67	6	75.00	16	55.17	^‡^	0.69	

^ Only asked among respondents that did not answer “Yes” to the previous response at 1 month. ^ψ^ Used a non-parametric Welch’s ANOVA due to not meeting the homogeneity of variance assumption. ^‡^ Fisher’s Exact Test was used due to small, expected cell counts. Thus, only a *p*-value is reported. ^b^ Statistically significant difference (*p* < 0.05) between low COI and high COI. ^c^ Statistically significant difference (*p* < 0.05) between middle COI and high COI.

**Table 3:** Healthcare utilization by time point and COI stratum: unadjusted descriptive and bivariate results.

Utilization	Window	Overall		Low COI		Middle COI		High COI		Statistic	*p*-Value	Statistically Significant Between COI Levels ^#^
		N	Mean/%	N	Mean/%	N	Mean/%	N	Mean/%			
Any follow-up visit ^1^; n (%)	0 to 14 days	152	74.88	31	60.78	25	73.53	95	81.20	7.87	0.0195 ^†^	^b^
	14 days to 1 month	115	62.16	26	54.17	24	75.00	64	61.54	3.55	0.17	
	1 to 2 months	73	41.48	20	44.44	17	56.67	35	35.00	4.75	0.09	
Time to first follow-up; mean (SD)	0 to 14 days	144	6.76 (5.00)	33	8.15 (4.18)	24	6.71 (3.75)	87	6.25 (5.51)	1.74	0.18	
Number of physician visits ^2^; mean (SD)	0 to 14 days	131	1.83 (1.29)	28	1.57 (0.92)	21	1.90 (1.45)	82	1.90 (1.36)	0.72	0.49	
Number of allied health visits ^3^; mean (SD)	0 to 14 days	45	2.07 (2.51)	7	1.00 (0.58)	8	1.38 (0.52)	29	2.45 (2.32)	2.11	0.13	
Number of CAM visits ^4^; mean (SD)	0 to 14 days	6	1.50 (1.22)	2	0.50 (0.71)	1	3.00	3	1.67 (1.15)	2.05	0.27	
Specialty seen; n (%) sports med	0 to 14 days	74	37.19	10	20.00	10	30.30	54	46.96	11.66	0.0029 ^†^	^b^
	14 days to 1 month	60	33.33	11	23.91	12	37.50	37	36.63	2.57	0.28	
	1 to 2 months	30	17.44	6	13.95	7	23.33	17	17.35	1.08	0.58	
Specialty seen; n (%) PT	0 to 14 days	16	8.25	1	2.04	1	3.13	14	12.50	^‡^	0.04	^b^
	14 days to 1 month	21	11.93	5	10.87	2	6.67	13	13.13	^‡^	0.68	
	1 to 2 months	22	12.79	5	11.36	3	10.34	14	14.29	^‡^	0.86	
Specialty seen; n (%) psychology	0 to 14 days	13	6.74	3	6.25	4	12.50	6	5.36	^‡^	0.40	
	14 days to 1 month	12	6.86	2	4.55	3	10.00	7	7.00	^‡^	0.59	
	1 to 2 months	8	4.73	1	2.38	2	6.90	5	5.15	^‡^	0.61	

Abbreviations: COI = Child Opportunity Index; CAM = complementary and alternative medicine; PT = physical therapy. ^1^ Consists of follow-up visits from any of these specialties: family physician, pediatrician, sports medicine physician, ophthalmologist, osteopathy, optometrist, physical therapy, occupational therapy, athletic trainer, psychology/mental health, chiropractor, myotherapy, acupuncture, and massage therapy. ^2^ Consists of follow-up visits from any of these specialties: family physician, pediatrician, sports medicine physician, ophthalmologist, and osteopathy. ^3^ Consists of follow-up visits from any of these specialties: optometrist, physical therapy, occupational therapy, athletic trainer, and psychology/mental health. ^4^ Consists of follow-up visits from any of these specialties: chiropractor, myotherapy, acupuncture, and massage therapy. ^†^ Significant linear trend (*p* < 0.05). ^‡^ Fisher’s Exact Test was used due to small, expected cell counts. Thus, only a *p*-value is reported. ^#^ For post hoc tests, pairwise comparisons of column proportions were computed. ^b^ Statistically significant difference (*p* < 0.05) between low COI and high COI.

**Table 4:** Family economic burden by time point and COI stratum: unadjusted descriptive and bivariate results.

Cost and Burden	Window	Overall		Low COI		Middle COI		High COI		Statistic	*p*-Value	Statistically Significant Between COI Levels ^#^
		N	Mean/%	N	Mean/%	N	Mean/%	N	Mean/%			
Total indirect costs; mean (SD)	0 to 14 days	190	283.98 (800.08)	48	202.04 (521.57)	34	235.97 (848.32)	107	338.65 (888.33)	0.56	0.57	
Workdays missed mean (SD)	0 to 14 days	202	2.17 (3.65)	50	3.38 (6.31)	34	2.09 (2.21)	117	1.70 (2.00)	3.81	0.02	^b^
Childcare days needed; mean (SD)	0 to 14 days	201	0.75 (2.00)	50	1.56 (2.70)	34	1.03 (2.83)	116	0.32 (1.04)	5.72 ^ψ^	0.01	^b^
School days missed; mean (SD)	1 month	181	2.77 (3.76)	45	3.11 (4.92)	32	2.63 (3.14)	103	2.67 (3.39)	0.24	0.79	
	2 months	163	2.81 (6.56)	40	4.23 (11.03)	29	3.10 (3.85)	93	2.10 (4.26)	1.51	0.22	
Any “other burden”; n (%) yes	0 to 14 days	15	7.35	3	5.88	2	5.88	10	8.47	^‡^	0.93	

^ψ^ Used a non-parametric Welch’s ANOVA due to not meeting the homogeneity of variance assumption. ^‡^ Fisher’s Exact Test was used due to small, expected cell counts. Thus, only a *p*-value is reported. ^#^ For post hoc tests, pairwise comparisons of column proportions were computed. ^b^ Statistically significant difference (*p* < 0.05) between low COI and high COI.

**Table 5:** Parental perception at 14 days and COI stratum: unadjusted descriptive and bivariate results.

Perception Item	Response Coding	Overall (N = 204)		Low COI		Middle COI		High COI		Statistic	*p*-Value
		N	%	N	%	N	%	N	%		
“My child quickly recovered”	Agree or strongly agree; n (%)	122	59.80	25	49.02	23	69.70	73	61.34	3.92	0.14
“Symptoms worse with activity”	Agree or strongly agree; n (%)	80	39.22	20	39.22	13	38.24	46	38.98	0.01	0.996
“My child has significant problems”	Agree or strongly agree; n (%)	20	9.80	5	9.80	4	11.76	10	8.47	^‡^	0.80
“Symptoms are constant”	Agree or strongly agree; n (%)	45	22.39	13	26.00	9	26.47	23	19.83	1.13	0.57
“Symptoms improved with time”	Agree or strongly agree; n (%)	183	89.71	42	82.35	31	91.18	109	92.37	3.96	0.14
“My child is exaggerating symptoms”	Agree or strongly agree; n (%)	7	3.45	3	5.88	1	2.94	3	2.56	^‡^	0.50

^‡^ Fisher’s Exact Test was used due to small, expected cell counts. Thus, only a *p*-value is reported.

**Table 6:** Multivariable models: association of COI with key outcomes.

Outcome	Model Type	COI Contrast	Effect Estimate	95% CI	*p*-Value	Covariates
PCSS trajectory	Mixed effects	Low vs. High	F = 2.53	-	0.11	age, sex, baseline PCSS, insurance, site, prior concussion
Symptom resolution by 1 month	Logistic	Low vs. High	Odds ratio = 0.887	0.32, 0.24	0.80	age, sex, insurance, site, prior concussion
Total indirect costs by 14 days	Two-part (logistic)	Low vs. High	Odds ratio = 0.687	0.35, 1.37	0.21	age, sex, insurance, site, prior concussion
		Middle vs. High	Odds ratio = 1.133	0.53, 2.43	0.41	age, sex, insurance, site, prior concussion
	Two-part (gamma regression with log link)	Low vs. High	Cost ratio = 0.9212	0.49, 1.73	0.80	age, sex, insurance, site, prior concussion
		Middle vs. High	Cost ratio = 0.5820	0.32, 1.07	0.08	age, sex, insurance, site, prior concussion
	Overall adjusted incremental cost	Low vs. High	Predicted mean difference = $19.91	−$12.20, $54.17	0.21	age, sex, insurance, site, prior concussion
		Middle vs. High	Predicted mean difference = −$69.47	−$104.75, −$35.17	<0.001	age, sex, insurance, site, prior concussion
Number of follow-up visits by 14 days	Poisson regression	Low vs. High	IRR = 0.7535	0.52, 1.11	0.13	age, sex, insurance, site, prior concussion
Follow-up visits by 14 days	Logistic	Low vs. High	Odds ratio = 0.362	0.16, 0.82	0.02	age, sex, insurance, site, prior concussion
Number of workdays missed by 14 days	Negative binomial	Low vs. High	IRR = 2.0109	1.28, 3.17	0.003	age, sex, insurance, site, prior concussion
Number of childcare days needed by 14 days	Negative binomial	Low vs. High	IRR = 4.1685	1.65, 10.51	0.003	age, sex, insurance, site, prior concussion

## Data Availability

The data presented in this study are available on reasonable request from the corresponding author. Access is restricted due to ethical and privacy considerations related to the clinical data. Requests will be evaluated to ensure protection of participant confidentiality and compliance with institutional requirements.

## Supplementary Materials

The following supporting information can be downloaded at: https://www.mdpi.com/article/10.3390/bs16081287/s1.

## Abbreviations

The following abbreviations are used in this manuscript:

mTBI	Mild Traumatic Brain Injury
PCSS	Post-Concussion Symptom Scale
COI	Child Opportunity Index
CAM	Complementary and Alternative Medicine
PT	Physical Therapy
